# A β‐1,2‐glucan‐associated glycoside hydrolase family 1 β‐glucosidase from *Streptomyces griseus*

**DOI:** 10.1002/pro.70255

**Published:** 2025-08-21

**Authors:** Haruto Kumakura, Sei Motouchi, Kaito Kobayashi, Miyu Inoue, Natsuki Kariuda, Hiroyuki Nakai, Masahiro Nakajima

**Affiliations:** ^1^ Department of Applied Biological Science, Faculty of Science and Technology Tokyo University of Science Noda Japan; ^2^ Artificial Intelligence Research Center National Institute of Advanced Industrial Science and Technology (AIST) Tokyo Japan; ^3^ Faculty of Agriculture Niigata University Niigata Japan

**Keywords:** glycoside glycosidase, β‐1,2‐glucan, β‐1,2‐glucanase, β‐1,2‐glucooligosaccharide, β‐glucosidase

## Abstract

β‐Glucosidases, major enzymes that release glucose from various natural compounds, are phylogenetically classified into glycoside hydrolase (GH) families. GH1 is the largest of these families. No β‐1,2‐glucan‐associated GH1 enzyme has been found, even though β‐1,2‐glucans are natural carbohydrates that are important for interaction between organisms and environmental adaptation. In this study, functional and structural analyses of a GH1 enzyme from *Streptomyces griseus* (SGR_2426 protein) were performed. SGR_2426 showed the highest hydrolytic activity toward *p*‐nitrophenyl β‐glucopyranoside among *p*‐nitrophenyl sugars. This enzyme showed hydrolytic activity toward β‐1,2‐glucooligosaccharides specifically among β‐linked glucooligosaccharides. A structure of the enzyme in complex with sophorose (β‐1,2‐glucodisaccharide) was obtained as a Michaelis complex. The six‐membered ring of the glucose unit at the reducing end of sophorose is positioned in a hydrophobic environment between Trp291 and Met171, while only residue Gln229 forms a hydrogen bond directly. Trp291 and Gln229 are proposed as candidates for the residues important for substrate specificity based on comparison with structurally characterized GH1 homologs. Mutational analysis of Trp291 and Gln229 suggested that Trp291 is important for substrate recognition but not for substrate specificity and that Gln229 is involved in substrate specificity. SGR_2426 is the first identified β‐1,2‐glucan‐associated β‐glucosidase in the GH1 family.

## 1 INTRODUCTION

Carbohydrates are important biomolecules that serve as energy sources and play crucial roles in cell wall components as cell skeletons, storage polymers such as starch, and interactions between organisms such as infection and symbiosis (Lenardon et al. 2010; Yamada et al. 2017; Zeeman et al. 2010). Carbohydrates have complex and diverse structures, corresponding to the wide variety of enzymes responsible for their synthesis and degradation. These enzymes have been classified into four main categories in the Carbohydrate‐Active enZYmes database: glycoside hydrolases (GHs), glycosyltransferases, polysaccharide lyases, and carbohydrate esterases; redox enzymes were recently added to this classification as a group called “auxiliary activity” (Drula et al. 2022; Henrissat and Davies 1997). The enzymes in this database are classified into families based on homology of their amino acid sequences. “GH family” is the most diverse category by number of families. As of Jul 2025, there are 194 GH families (including eight deleted families).

Glucose is one of the representative monosaccharides and forms glucans such as cellulose, laminarin, and starch. Numerous exo‐ and endo‐type enzymes that act on these glucans have been reported, including cellulases and cellobiohydrolases (β‐1,4‐linkages), β‐1,3‐glucanases, amylases, and glucoamylases (α‐1,4‐linkages), and their functions, structures, and molecular mechanisms have been studied extensively (Janeček et al. 2014; Linton 2020). However, there is much less knowledge regarding enzymes that act on β‐1,2‐glucans compared with other enzyme groups.

β‐1,2‐Glucan was first discovered in the 1940s as a polymer secreted by *Agrobacterium*, a phytopathogen that causes crown gall on plant roots (McIntire et al. 1940; McIntire et al. 1942). The compound was later proved to be a cyclic glucan, and its physiological functions were reported (Putman et al. 1950; Zevenhuizen and Scholten‐Koerselman 1979). Cyclic β‐1,2‐glucans in *Ensifer fredii* JCM20967 (formerly *Sinorhizobium* sp. strain NGR234) are important for symbiosis with plants. Cyclic β‐1,2‐glucans are also responsible for evasion of host defense systems during infection by *Brucella abortus* (Arellano‐Reynoso et al. 2005; Gay‐Fraret et al. 2012). Genes encoding cyclic β‐1,2‐glucan synthases were identified in *Rhizobium radiobacter* (*Agrobacterium tumefaciens*), *Ensifer meliloti* (*Sinorhizobium meliloti*), and *B. abortus* (Castro et al. 1996; Iannino et al. 1998; Roset et al. 2004; Zorreguieta and Ugalde 1986). The detailed molecular mechanism of cyclic β‐1,2‐glucan synthesis has been elucidated (Ciocchini et al. 2007; Sedzicki et al. 2024). However, prior to 2014, only a few studies reported enzymatic activities toward β‐1,2‐glucans originating from a bacterium and some filamentous fungi without gene identification (Kitahata and Edagawa 1987; Mendoza and Amemura 1983; Nakajima 2023; Reese et al. 1961).

**FIGURE 1:**
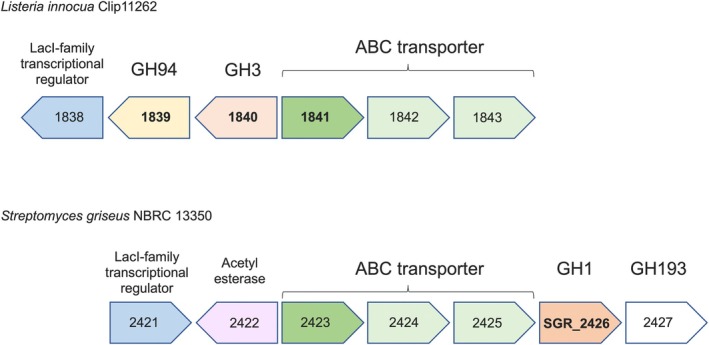
Schematic representation of gene clusters related to the metabolism of Sop_n_s in *L. innocua* and *S. griseus* genomes. Locus tags without “Lin_” or “SGR_” are shown in arrowheads. The genes coding characterized proteins and the target protein in this study are shown in bold.

More recently, a novel glycoside phosphorylase that acts on β‐1,2‐glucooligosaccharides (Sop_n_s, where *n* is the degree of polymerization; “S” is an abbreviation for sophorooligosaccharides, an alternative name for β‐1,2‐glucooligosaccharides) was discovered from *Listeria innocua* (protein Lin1839 in GH94) (Figure 1) (Nakajima et al. 2014), which led to the development of a large‐scale production method of β‐1,2‐glucans (Abe et al. 2015; Kobayashi et al. 2019; Nakajima et al. 2021). Identification of β‐1,2‐glucanases (SGLs) from a bacterium and a filamentous fungus by utilizing these polysaccharides led to the establishment of GH144 and GH162 families, respectively (Abe et al. 2017; Tanaka et al. 2019). Recently, OpgG and OpgD from *Escherichia coli*, osmoregulated periplasmic glucan biosynthesis proteins with unknown biochemical functions, were found to be SGLs, which established a new GH family, GH186 (Motouchi et al. 2023). In addition, an enzyme cyclizing a linear β‐1,2‐glucan to produce a cyclic β‐1,2‐glucan was found as a GH189‐establishing member. An enzyme that produces an α‐1,6‐cyclized β‐1,2‐glucan was also found from GH186 recently (Motouchi et al. 2024; Tanaka et al. 2024).

Along with the findings of new GH families, a novel exo‐type GH144 enzyme that releases a sophorose unit from Sop_n_s was discovered (Shimizu et al. 2018), and a novel transglycosylase from GH35 that acts on β‐1,2‐glucosidic bonds was discovered by exploring an SGL gene cluster of a Gram‐negative bacterium (Kobayashi et al. 2022). Furthermore, in the gene cluster of the *lin1839* gene in *L. innocua*, a sugar‐binding subunit of an ABC transporter that binds to Sop_n_s was found, and a GH3 enzyme was evidenced to be a β‐glucosidase (BGL) preferring Sop_n_s as substrates (proteins Lin1841 and Lin1840, respectively) (Abe et al. 2018; Nakajima et al. 2014; Nakajima et al. 2016) (Figure 1). These findings suggest that the products of the gene cluster can metabolize β‐1,2‐glucans and/or Sop_n_s. A GH3 homolog from *Bacteroides thetaiotaomicron* exhibited the same linkage position specificity as Lin1840 (Ishiguro et al. 2017). Tertiary structures of these newly discovered enzymes are now available, and their structure–function relationships have been clearly explained (Abe et al. 2017; Abe et al. 2018; Ishiguro et al. 2017; Kobayashi et al. 2022; Nakajima et al. 2016; Nakajima et al. 2017; Shimizu et al. 2018; Tanaka et al. 2019).

BGLs, which are essential enzymes for releasing glucose exolytically from the non‐reducing end, mainly belong to GH1 and GH3 families, and some BGLs are found in GH2, GH5, GH30, GH116, GH131, GH175, and GH180 (Broeker et al. 2018; Ferrara et al. 2014; Helbert et al. 2019; Schröder et al. 2015; Shen et al. 2019). The GH1 family is so diverse in amino acid sequences that regions in substrate pockets recognizing moieties lacking a non‐reducing end glucose unit in a substrate show wide structural variety. However, no GH1 BGL involved in the metabolism of β‐1,2‐glucans and/or Sop_n_s has been reported. *Streptomyces griseus*, a bacterium famous for producing secondary metabolites (Lacey and Rutledge 2022), has a homolog of the Sop_n_s‐binding subunit of the ABC transporter from *L. innocua* (Figure 1). The gene cluster encoding this protein contains SGR_2426, a GH1 protein, and SGR_2427 protein belonging to GH193 (Figure 1). Therefore, we hypothesized that SGR_2426 is involved in the metabolism of β‐1,2‐glucans and Sop_n_s. SGR_2426 has only been analyzed in screens for cellulose‐degrading enzymes, and its activity toward lactose, cellobiose, and xylobiose has been roughly investigated, though no proper quantitative data are available (Heins et al. 2014). In this study, through functional and structural analyses, we reveal that SGR_2426 is a β‐1,2‐glucan‐associated BGL that acts preferentially on Sop_n_s as substrates.

## 2 RESULTS

### 2.1 General properties of SGR_2426r

SGR_2426 does not contain a predicted *N*‐terminal signal peptide. The recombinant SGR_2426 (SGR_2426r) His‐tagged at the *C*‐terminus was successfully expressed in *E. coli* and purified using a nickel affinity chromatography column (Figure S1a, Supporting Information). The purified enzyme showed the highest hydrolytic activity toward *p*‐nitrophenyl‐β‐d‐glucopyranoside (pNP‐β‐Glc) among pNP‐sugars tested. The enzyme showed approximately 31% and 27% relative activity toward pNP‐β‐d‐fucopyranoside (pNP‐β‐d‐Fuc) and pNP‐β‐d‐galactopyranoside (pNP‐β‐Gal), respectively, in the presence of 5 mM substrate (Table 1). Thus, pNP‐β‐Glc was used as the substrate in investigations of pH and temperature profiles of enzyme activity (Figure S1b–e). The enzyme showed high activity at pH 5.5–7.5. At pH 6.5, the enzyme showed similar activity in the presence of either MOPS or Bis‐Tris buffer, but MES buffer had some effect on the activity. Large differences in activities between buffers with the same pHs were also found at pH 5.5, 6.5, and 9.0, which may be due to buffer effect. SGR_2426r showed sufficient stability at pH 7.5–9.0, while the enzyme was not stable at pH 6.5. Thus, the enzyme was diluted using 50 mM MOPS (pH 7.5) and an assay of kinetics was performed at pH 6.5. Substrates of SGR_2426r may stabilize the enzyme at pH 5.5–6.5. SGR_2426r showed its highest activity at 30°C and was stable at up to 30°C, which is consistent with the optimum growth temperature of *S. griseus* (Gunjal and Bhagat 2021). It should be noted that there is approximately 20% and 40% relative activity at 0 and 10°C, respectively, which implies that the enzyme is cold‐adaptive as in the case of Antarctic *Marinomonas* sp. ef1 (Gourlay et al. 2024).

**TABLE 1:** Kinetic parameters of SGR_2426r.

	*V*_max_ (U/mg)	*K*_m_ (mM)	*V*_max_/*K*_m_ (U/mg/mM)	Linear regression for *V*_max_/*K*_m_ ^d^	*V*_max2_ (U/mg)	*K*_m2_ (mM)	*V*_max2_/*K*_m2_ (U/mg/mM)
Wild‐type							
pNP‐β‐Glc	28 ± 1	1.0 ± 0.1	28 ± 2				
pNP‐β‐d‐Fuc	4.6 ± 0.3	0.16 ± 0.1	29 ± 3.1		21 ± 10	27 ± 20	0.79 ± 0.26
pNP‐β‐Gal	13 ± 1	5.8 ± 0.3	2.3 ± 0.1	1.9 ± 0.1			
Sop_2_	36 ± 2	17 ± 2	2.0 ± 0.1	1.8 ± 0.1			
Sop_3_	35 ± 2	19 ± 2	1.9 ± 0.1	1.6 ± 0.1			
Sop_4_	24 ± 2	11 ± 2	2.2 ± 0.2	1.9 ± 0.2			
Sop_5_	–^a^	–^a^	(3.4)	3.8 ± 0.3			
Lam_2_	–^b^	–^b^	0.089 ± 0.006	0.079 ± 0.002			
Cel_2_	(<0.01)^c^						
Gen_2_	(<0.01)^c^						
Lam_3–5_	(<0.01)^c^						
W291E							
Sop_2_	4.7 ± 1.1	36 ± 11	0.13 ± 0.01	0.11 ± 0.01			
Lam_2_	(0.0042)^c^						
Cel_2_	(<0.001)						
Gen_2_	(<0.001)						
Q229N							
Sop_2_	2.1 ± 0.4	33 ± 9	0.064 ± 0.005	0.057 ± 0.003			
Lam_2_	–^b^	–^b^	0.020 ± 0.002	0.017 ± 0.001			
Cel_2_	(<0.001)						
Gen_2_	(<0.001)						

^a^ The kinetic values were not determined, although the curve was fitted with the plots. The value in a parenthesis represents the slope when the plots (up to 2 mM) were applied to linear regression.

^b^ These values are not accurate because of the high *K*_m_ value. Thus, the calculated values are provided here for reference only. Lam_2_ (WT), *V*_max_ = 8.4 ± 4.2, *K*_m_ = 95 ± 54; Lam_2_ (Q229N), *V*_max_ = 1.8 ± 0.8, *K*_m_ = 92 ± 42.

^c^ Specific activity in the presence of 5 mM substrate.

^d^ Linear regression was performed using the data up to 1.5 mM for pNP‐Gal (WT), 2 mM for Sop5 (WT), 15 mM for Lam_2_ (WT and Q229N), and 3 mM for the other samples as described in section 4.

### 2.2 Substrate specificity of pNP‐sugars

**FIGURE 2:**
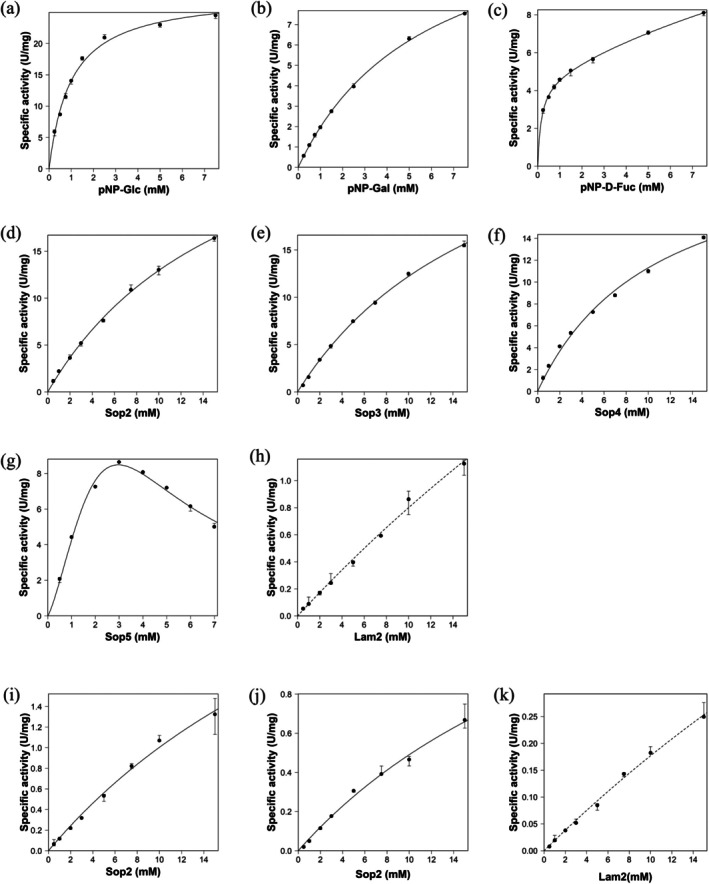
Kinetic analysis of SGR_2426r. Medians of the triplicate experiments are used for plots. The other data are used for error bars. (a–h) The wild‐type, (i) W291E mutant, (j–k) Q229N mutant. (h, k) The fitting curves are depicted as dashed lines because the errors of *V*_max_ and *K*_m_ values are large.

SGR_2426r showed comparable hydrolytic activity toward pNP‐β‐Glc, pNP‐β‐d‐Fuc, and pNP‐β‐Gal, and therefore kinetic analysis was performed using these substrates (Table 1 and Figure 2a–c). Data for pNP‐β‐Glc and pNP‐β‐Gal were regressed with the Michaelis–Menten equation. Contrarily, data for pNP‐β‐d‐Fuc was regressed with a biphasic equation rather than the Michaelis–Menten equation, which suggests a second substrate molecule participates in the reaction. Thus, a transglycosylation reaction could occur on pNP‐β‐d‐Fuc. The *V*_max_ value for pNP‐β‐Glc (28 U/mg) was approximately twice the value of pNP‐β‐Gal (13 U/mg). The *K*_m_ value for pNP‐β‐Glc (1.0 mM) was approximately 6 times lower than that for pNP‐β‐Gal (5.8 mM). Consequently, the *V*_max_/*K*_m_ value for pNP‐β‐Glc was over 10 times higher than that for pNP‐β‐Gal. In the case of pNP‐β‐d‐Fuc, although the *V*_max_ and *K*_m_ values were remarkably different from the *V*_max2_ and *K*_m2_ values, hydrolytic activity toward pNP‐β‐Glc was apparently higher than that for pNP‐β‐d‐Fuc according to the plots. These results suggest that SGR_2426r is a β‐glycosidase with a preference for β‐glucosides.

### 2.3 Substrate specificity of glucooligosaccharides

Because SGR_2426r preferred a β‐glucoside among tested pNP‐sugars, its substrate specificity toward β‐linked glucooligosaccharides was investigated. In the presence of 5 mM glucodisaccharide, the specific activity was the highest toward Sop_2_ (sophorose, β‐1,2‐glucodisaccharide). The enzyme exhibited approximately 5.2% of the specific activity toward Lam_2_ (here, laminarioligosaccharides, β‐1,3‐glucooligosaccharides, are represented as Lam_n_, where *n* is the degree of polymerization) compared to Sop_2_ (Table 1 and Figure 2d–h). The enzyme showed very low specific activities (<0.001 U/mg) toward cellobiose (Cel_2_, β‐1,4‐glucodisaccharide) and gentiobiose (Gen_2_, β‐1,6‐glucodisaccharide). Thus, kinetic analysis toward Sop_n_s and Lam_2_ was performed. *V*_max_ and *K*_m_ values and the resulting *V*_max_/*K*_m_ values were comparable for Sop_2–4_ (Table 1 and Figure 2d–f). While the *K*_m_ values for them were larger than a usual range of typical BGLs, the *V*_max_/*K*_m_ values are within the range (Opassiri et al. 2004), suggesting that Sop_2–4_ are actual substrates of SGR_2426. Although kinetic parameters for Sop_5_ were not determined by fitting the plots, unfortunately, the pattern of the plots could explain substrate inhibition or transglycosylation. Specific activity at low concentrations was a similar level to those of Sop_2–4_ (Table 1 and Figure 2g). This result suggests that Sop_5_ is also an actual substrate for SGR_2426 as well as Sop_2–4_.

Next, kinetic analysis was performed with Lam_2_ as the substrate (Table 1 and Figure 2h). The *V*_max_ value was comparable to those for Sop_2–4_. The *V*_max_ and *K*_m_ values for Lam_2_ were approximately 5 times lower and 5.5 times higher than those for Sop_2_, although their error values were somewhat high. The resulting *V*_max_/*K*_m_ value was thus >20 times smaller than that for Sop_2_, suggesting that Lam_2_ is not a natural substrate of the enzyme. In addition, the enzyme showed low activity toward Lam_3–5_ (<0.001 U/mg). Overall, these results suggest that SGR_2426 is specific for the β‐1,2‐linkage in glucooligosaccharides.

### 2.4 Structure of SGR_2426r

**FIGURE 3:**
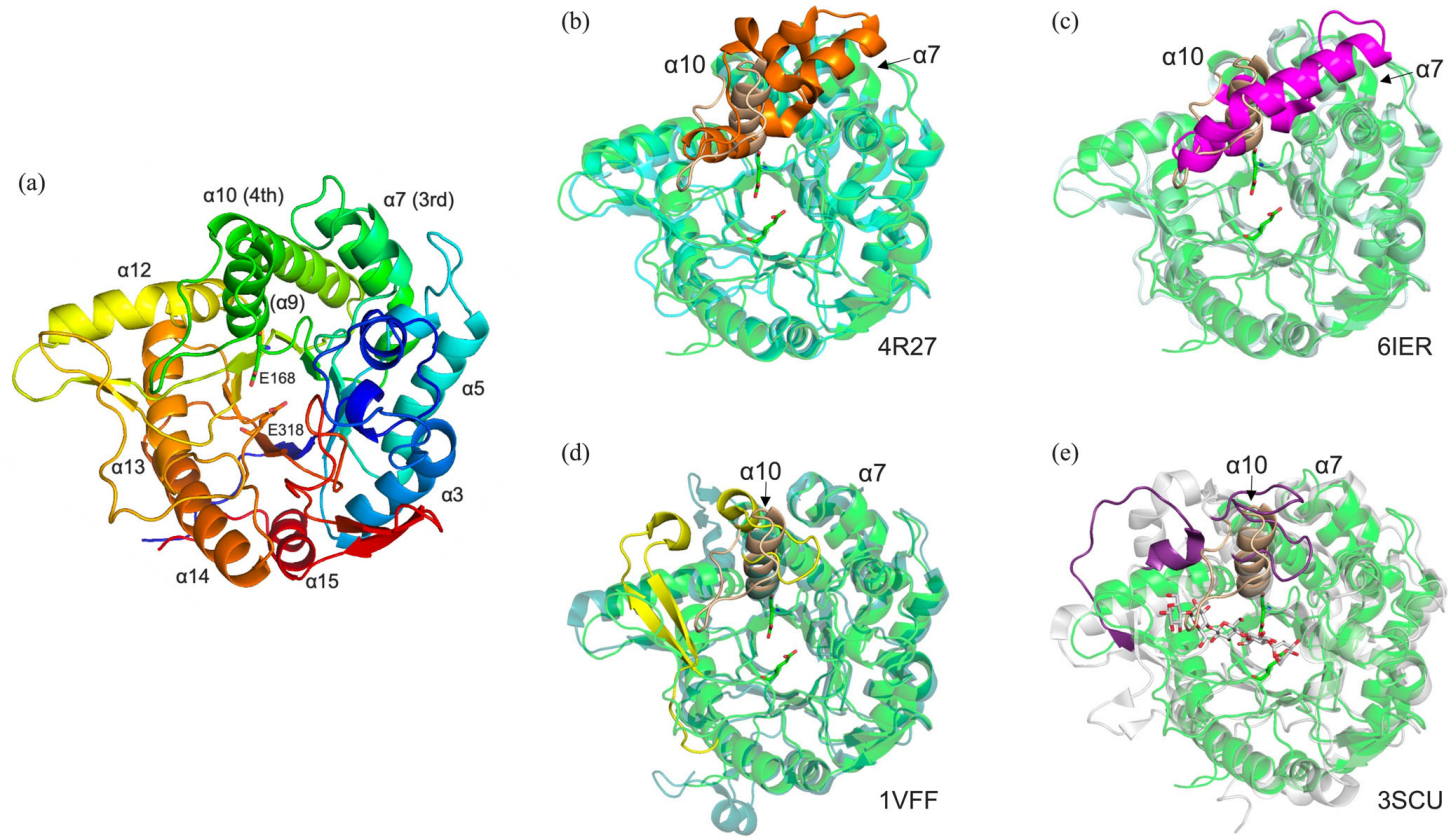
Overall structure of SGR_2426r. (a) The ligand‐free structure of SGR_2426r is shown as a rainbow cartoon. The PDB ID of the ligand‐free structure of SGR_2426r is 8KAP. The *N*‐terminal end is colored blue. α‐Helices in the (β/α)_8_‐barrel are labeled. The inserted α‐helix (α9) is labeled with a parenthesis. The numbers by α‐helices correspond to the numbers in the multiple sequence alignment shown in Figure S2. (b–e) Superimposition of SGR_2426r with glycoside hydrolase family 1 (GH1) homologs. The superimposed proteins are the top four homologs retrieved from the KEGG database after a BLASTP search using SGR_2426 as the query. SGR_2426r is shown as a semi‐transparent green cartoon, except for residues 169–195, which are shown as a light‐brown cartoon. The corresponding regions in the superimposed proteins are shown as orange, magenta, yellow, and purple cartoons, respectively. The other parts are shown as semi‐transparent cyan, light‐cyan, blue–green, and light‐blue–green cartoons, respectively. PDB IDs are shown at the bottom‐right of the images (4R27, BGL167 from *Microbacterium* sp. Gsoil167; 6IER, a protein from an uncultured bacterium; 1VFF, β‐glycosidase from *Pyrococcus horikoshii*; 3SCU, BGlu1 from *Oryza sativa* Japonica Group). α‐Helices (α7 and α10) in SGR_2426r are labeled.

A ligand‐free X‐ray crystal structure of SGR_2426r was determined at 2.20‐Å resolution (Table S1). There are four chains in the asymmetric unit, each with almost the same configuration (root‐mean‐square deviation <0.25 Å) (Figure 3a). However, the biological assembly was judged to be a monomer according to the PISA server (https://www.ebi.ac.uk/msd-srv/prot_int/pistart.html) (Krissinel and Henrick 2007). Similar to other structurally characterized GH1 enzymes, the enzyme possesses a (β/α)_8_‐barrel domain. There are, however, obvious differences in the shape of the substrate pocket compared with structurally characterized close homologs (Akiba et al. 2004; Park et al. 2015; Seshadri et al. 2009), especially in the α‐helix region of residues 169–195 (Figure 3b–e). This α‐helix (light‐brown in Figure 3b–e) is inserted between the 3rd and 4th α‐helices of the TIM‐barrel (Figures 3 and S2). Amino acid sequences and structures of this region are drastically diversified among GH1 enzymes, which are probably related to substrate specificity (as discussed later).

### 2.5 Complex structure with Sop_2_

**FIGURE 4:**
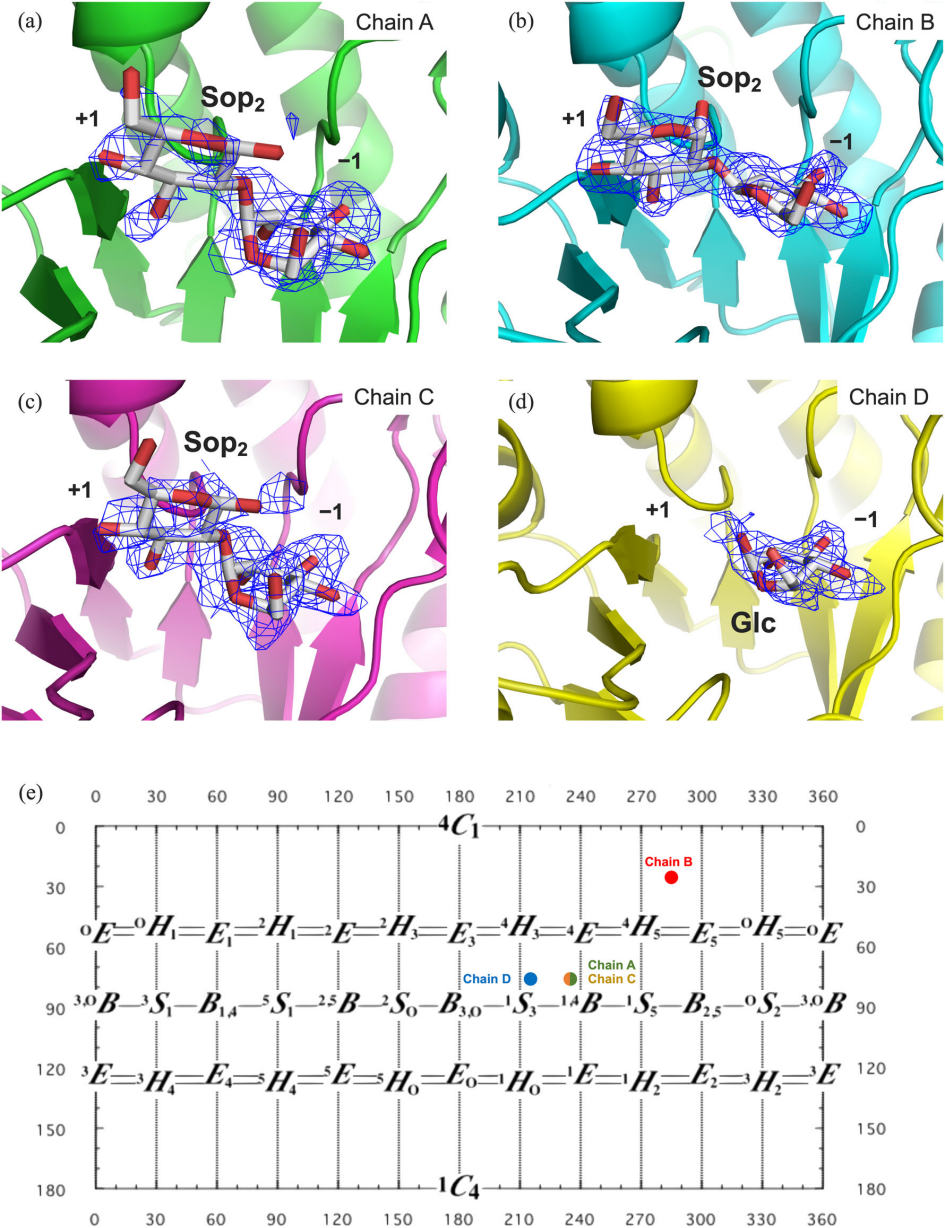
Conformations of substrates in the catalytic pockets of SGR_2426r. (a–d) Electron densities of Sop_2_ (a–c) and Glc (d) in the catalytic pockets of the SGR_2426r–Sop_2_ complex. Chains (a–d) of SGR_2426r (PDB ID, 9U98) are shown as green, cyan, magenta, and yellow cartoons, respectively. Sop_2_ and Glc are shown as white sticks. *F*_o_ − *F*_c_ maps are shown at the 3σ contour level as blue meshes. The numbers indicate subsite positions. (a–c) The glucose moieties at the reducing end perhaps contain both anomers in solution, although only one anomer is fitted as a model in each subunit. (e) Six‐membered ring puckering of the Glc moieties at subsite −1 in the complex of SGR_2426r with Sop_2_. Calculation of Cremer–Pople parameters was performed using the site created by Dr. Fushinobu (http://enzyme13.bt.a.u‐tokyo.ac.jp/CP/). The panel is a figure that the results obtained from the four chains are integrated. Each of the plots is manually placed based on each result. Because the plots for Chain A and Chain C are located at almost the same position, the plots are represented as hemispheres.

To obtain a complex structure with a substrate, residues Glu168 and Glu318, which are candidates for an acid/base and a nucleophile involved in the catalytic reaction, were replaced with glutamine and glycine residues, respectively. Both mutants showed drastically decreased hydrolytic activity toward pNP‐β‐Glc compared with the wild type (data not shown). Because soaking with usual concentrations of Sop_2_ (approximately 10–20 mM) did not yield sufficient electron density of Sop_2_, crystals of the E318G mutant prepared without Sop_2_ were soaked in a cryoprotected solution containing 500 mM Sop_2_. The overall structure of the complex is almost the same as that of the ligand‐free enzyme, although the space groups of the crystals were different between the ligand‐free and the complex structures. However, conformations of glucose (Glc) moieties at subsite −1 (subsite is a nomenclature for substrate binding sites, see https://www.cazypedia.org/index.php/Sub-site_nomenclature for details) (Davies et al. 1997), electron density of Glc moieties at subsite +1, and B‐factors for parts of the substrate pocket vary between the four molecules (chains A–D) in the asymmetric unit (Figures 4a–d, S3, and S4). In addition, two other Sop_2_ molecules were observed at the interface of chains A and C clearly in the asymmetric unit (Figure S3). Binding of these Sop_2_ molecules might be an artifact because the enzyme was judged to be a monomer by PISA analysis.

To assess if the substrate binding mode is plausible for the reaction, six‐membered ring puckering of the Glc moieties at subsite −1 was analyzed using the Cremer–Pople model (Cremer and Pople 1975). In chain B, the electron density of Sop_2_ was clearly observed and the Glc moiety at subsite −1 adopted a chair conformation (^4^ *C*_1_) (Figure 4b). This conformation is a state immediately after substrate binding, which is not suitable for the reaction because distortion of the six‐membered ring is needed to reach the transition state. In chains A and C, the electron densities of the Glc moieties at subsite +1 are slightly less observed than that in chain B (Figure 4a–c), and the Glc moieties at subsite −1 adopt distorted conformations around ^1^ *S*_3_, ^1,4^ *B*, and ^4^ *E* (Figure 4e). In chain D, the Glc moiety at subsite −1 adopts a skewed conformation (^1^ *S*_3_) (Figure 4e), which is often observed in the Michaelis complex in GH enzymes (Nakajima et al. 2016; Tanaka et al. 2019). Furthermore, the Glc moiety at subsite +1 is almost completely disordered (Figure 4d). Based on the fact that both ^1^ *S*_3_ and ^4^ *C*_1_ conformations of substrates at subsite −1 are structurally available in GH1 BGLs, reaction itineraries are suggested to proceed via a ^1^ *S*_3_ → ^4^ *H*_3_ → ^4^ *C*_1_ pathway (Sansenya et al. 2011; Seshadri et al. 2009; Verdoucq et al. 2004). Thus, the three different conformations obtained in this study are consistent with the suggested itinerary.

The clarity of electron densities at subsite +1 decreases as the reaction proceeds according to this itinerary (Figure 4a–d). To understand the reason for this difference in clarity, the B‐factor of each chain was visualized (Figure S4). Chains A–C showed similar B‐factors (Table S1). Nevertheless, regions with large B‐factors in Chain B are larger than those in Chains A and C (Figure S4a–c). Unlike in these three chains, there are three disordered regions in chain D (residues 182–188, 232–243, and 271–282) (Figure S4d). Superimposition of chains B and A, A and C, and C and D suggests that the extent of deviations between chains corresponds to the conformational changes of the Glc moieties at subsite −1 (Figure S5a–c). Five deviated regions (designated as regions 1–5 in Figure S5a) are found. Regions 2–4 are the same as the distorted regions in Chain D. Regions 1 and 5 are additionally found as deviated regions (Figures S4 and S5a–c). Region 1 interacts with the Glc moiety at subsite −1 and region 5 forms the entrance of the catalytic pocket along with the regions (residues 182–188 and 271–282). The five regions are found to exhibit relatively larger fluctuations (root mean square fluctuation, RMSF) according to the molecular dynamics (MD) simulation of the ligand‐free structure (Figure S5d). The fluctuation of region 5 reduced when the complex with Sop_2_ was used for the simulation, which may be due to binding to the substrate.

### 2.6 Substrate recognition in the Sop_2_ complex

**FIGURE 5:**
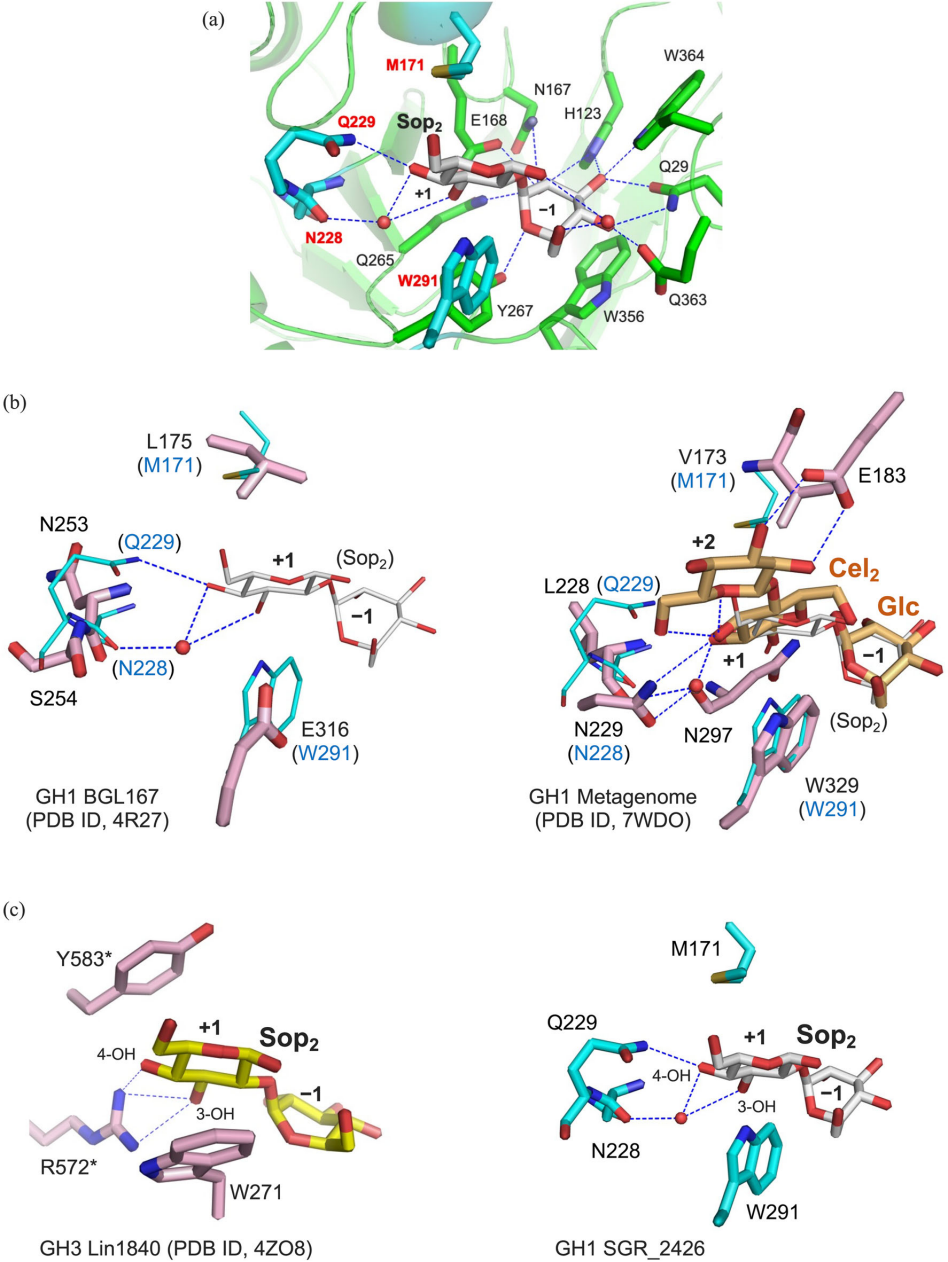
Comparison of binding modes of substrates between GH1 enzymes. Hydrogen bonds are shown as blue dashed lines. Distances up to 3.5 Å are regarded as hydrogen bonds. A water molecule is shown as a sphere. Main‐chain atoms that are not involved in substrate recognition are omitted for visuality. (a) Binding mode of Sop_2_ in the SGR_2426r–Sop_2_ complex. Chain A of the complex is used for visualization (PDB ID, 9U98). The main‐chain is shown as a green cartoon. The numbers beside the Glc moieties are subsite positions. Recognition residues for subsites −1 and +1 are shown as green and cyan sticks, respectively, and are labeled with black plain and red bold letters, respectively, for SGR_2426r. (b) Comparison of binding modes of ligands with BGL167 (left) and MeBglD2 (right). SGR_2426r (PDB ID, 9U98) is superimposed with BGL167 (from *Microbacterium* sp. Gsoil167) and MeBglD2 (from a metagenome). SGR_2426r is shown as thin sticks in the same color as (a). Residues in the other enzymes are shown as light pink sticks. The labels for residues in SGR_2426r are shown in blue. Sop_2_ molecules are shown as thin white sticks. Parentheses indicate labels for SGR_2426r. (right) Cel_2_ and Glc are shown as light‐brown sticks. Subsite positions are labeled with numbers. (c) Comparison of binding modes of Sop_2_ between GH3 BGL (Lin1840, PDB ID 4ZO8, left) and SGR_2426r–Sop_2_ complex (PDB ID, 9U98, right). (left) Residues recognizing the Glc moieties at the subsites +1 and Sop_2_ molecule are shown as pink and yellow sticks, respectively, for Lin1840. Asterisks represent that the residues are derived from another subunit in the dimer. (right) SGR_2426r is shown in the same way as (b).

The Glc moiety at subsite −1 is firmly recognized by multiple residues (Gln29, His123, Asn167, Tyr267, Gln363, Trp364) through hydrogen bonds. In contrast, only a single hydrogen bond is observed at subsite +1, involving Gln229 (Figure 5a). The main chain of Asn228 indirectly recognizes the substrate through a water molecule. The six‐membered ring of the Glc moiety at subsite +1 is sandwiched between Met171 and Trp291 hydrophobically. These observations suggest that substrate recognition at subsite +1 is loose, being consistent with the disordered electron density at subsite +1 in Chain D. However, the anomeric hydroxy group at subsite +1 faces solvent, suggesting that Sop_2_ binds correctly to the enzyme in the crystal.

### 2.7 Mutational analysis

To understand the substrate preference of SGR_2426r in detail, W291E and Q229N mutants were investigated based on the observations described below. GH1 BGL from *Mycobacterium* sp. Gsoil167 is an enzyme that hydrolyzes glucosides derived from a triterpenoid saponin (Rb1) containing Sop_2_ and Gen_2_ (Park et al. 2015). Non‐reducing end Glc moieties in Sop_2_ and Gen_2_ are hydrolyzed by this enzyme. An E316W mutant loses hydrolytic activity toward the β‐1,6‐glucosidic linkage. E316 corresponds to Trp291 in SGR_2426, implying the importance of this residue for specificity toward both β‐1,2‐ and β‐1,6‐linkages (Figure 5b, left). Meanwhile, Asn253 and Ser254, corresponding to Gln229 in SGR_2426, seem not to be involved in substrate recognition because their side chains face the opposite side compared with Gln229 in SGR_2426. Thus, the W291E mutant was prepared to elucidate the roles of W291.

Trp291 in SGR_2426 is conserved in a GH1 BGL from a metagenome (Genbank accession number, BAV69317.1); however, the latter enzyme shows similar activity toward β‐linked glucodisaccharides (Sop_2_, Lam_2_, Cel_2_, and Gen_2_), unlike SGR_2426 (Matsuzawa et al. 2022). Gln229 in SGR_2426 is also conserved (as a chemically similar residue) in the enzyme from the metagenome (Figure 5b, right). Thus, Q229N was also prepared to elucidate the roles of Q229.

Consequently, W291E showed a remarkable decrease in activity on Sop_2_ and Lam_2_ (Table 1 and Figure 2i). The *V*_max_ value decreased approximately 5 times and the *K*_m_ value increased approximately twice that of the wild‐type. No activity on Cel_2_ nor Gen_2_ was detected. Only a weak activity was detected on Lam_2_. The ratio of specific activity between Sop_2_ and Lam_2_ in the presence of 5 mM substrate was at a level similar to that of the wild‐type enzyme. This result suggests that W291 is important for substrate recognition but does not contribute to substrate preference. The hydrolytic activity of the Q229N mutant toward Sop_2_ was also reduced remarkably, as well as that of the W291E mutant (Table 1 and Figure 2j,k). However, activity toward Lam_2_ was retained apparently compared to the W291E mutant, suggesting that Q229 contributes to the substrate preference of SGR_2426r.

## 3 DISCUSSION

SGR_2426 was previously investigated as a possible cellulose‐degrading enzyme (Heins et al. 2014). Among *Streptomyces* species, SCO1059 and SCO7558 from *Streptomyces coelicolor* A3(2), a protein from *Streptomyces rochei* A2 (accession number, CAA52344.1), SCAB_2391 and SCAB_57721 from *Streptomyces scabiei* 87.22, and BgluS from *Streptomyces* sp. RW2 (accession number, UUY85975.1) were reported to be GH1 family enzymes. However, only Cel_2_ and glycosides were investigated as substrates (Deflandre et al. 2020; Gu et al. 2013; Heins et al. 2014; Jourdan et al. 2018; Mastromei et al. 1992; Mei et al. 2021); thus, natural substrates have not been fully investigated. These homologs, except the *S. rochei* A2 protein, are far from SGR_2426 phylogenetically (Figure S6). Characteristics of close homologs of SGR_2426 other than the *S. rochei* A2 protein have not been investigated. A GH1 BGL from *Talaromyces amestolkiae* is annotated as β‐1,2‐glucosidase in the CAZy database (Drula et al. 2022). Although the enzyme acts on a β‐1,2‐glucosidic linkage in glucosides, it also shows comparable hydrolytic activity on cellooligosaccharides (Méndez‐Líter et al. 2020). This fact suggests whether the BGL is β‐1,2‐glucan‐associated or not is controversial. Os3BGlu6 from rice (*Oryza sativa*) is reported to be a GH1 family BGL that prefers β‐1,2‐ and β‐1,3‐linked glucodisaccharides as substrates (Seshadri et al. 2009). However, Os3BGlu6 preferentially acts on pNP‐β‐d‐Fuc over pNP‐β‐Glc, indicating that the enzyme is actually a β‐d‐fucosidase (Seshadri et al. 2009).

This study revealed that SGR_2426r had comparable hydrolytic activity toward Sop_2–5_ among the tested glucooligosaccharides in the presence of up to 2 mM substrate and that the enzyme showed low affinity (a high *K*_m_ value) for Lam_2_ (β‐1,3) and very low activities toward Cel_2_ (β‐1,4) and Gen_2_ (β‐1,6) (Table 1). These results suggest that SGR_2426 is not an enzyme for cellobiose degradation, but is the first identified GH1 enzyme acting on Sop_n_s as natural substrates. It should be noted that SGR_2426 prefers Sop_2_ over Lam_2_ much more strongly than Lin1840 and BT_3567, GH3 β‐1,2‐glucan‐associated enzymes hydrolyzing Sop_2_ and Lam_2_ with comparable velocity (Ishiguro et al. 2017; Nakajima et al. 2016).

**FIGURE 6:**
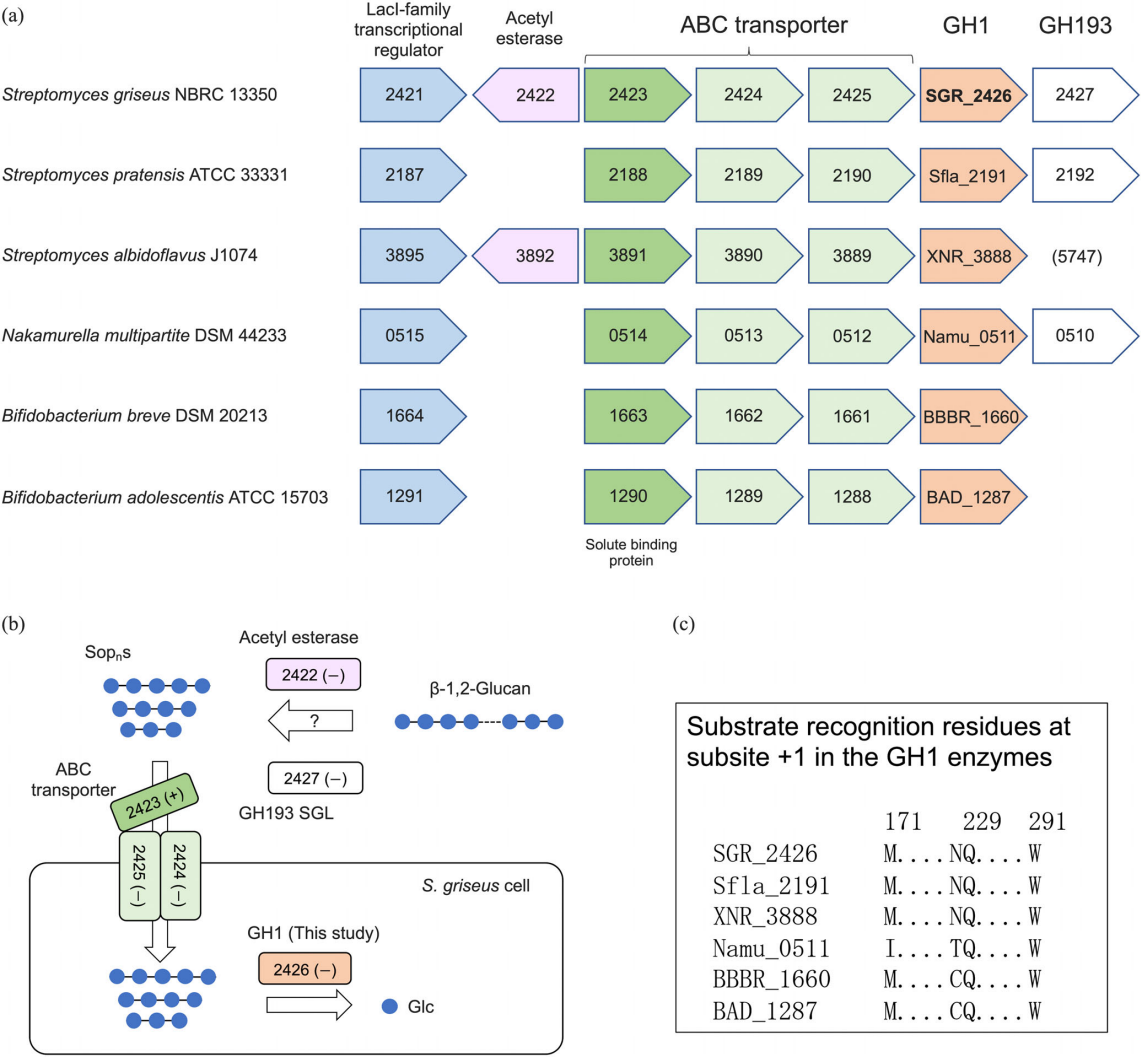
Schematic representation of gene clusters containing homologs of gene *SGR_2426* and β‐1,2‐glucan metabolism. (a) Gene clusters containing homologs of gene *sgr_2426*. Annotations of proteins are based on information in the KEGG database (https://www.kegg.jp/kegg/kegg2.html). Annotation of GH193 is based on the previous study (Nakajima et al. 2025). Parentheses indicate that the homologs are not in the gene clusters of the *sgr_2426* gene homologs. Directions of arrowheads correspond to directions of genes. (b) Speculated β‐1,2‐glucan metabolism in *S. griseus*. Glc units are depicted based on Neelamegham et al. (2019). (+) and (−) represent proteins with and without a predicted *N*‐terminal signal, respectively. A question represents that a degradation step is not supported by the results of prediction of *N*‐terminal signal peptides. (c) Comparison of residues participating in recognition at subsite +1 between the six GH1 enzymes. The residues are extracted from a multiple sequence alignment (Data S2).

The gene cluster that contains the SGR_2426‐encoding gene also encompasses ABC transporter homolog genes (*sgr_2423*–*2425*) (Figure 6a). SGR_2425, annotated as a solute‐binding protein, is a close homolog of Lin1841 from *L. innocua*, a binding subunit for Sop_3–5_ in an ABC transporter (Abe et al. 2018); SGR_2425 is expected to have a similar substrate preference to Lin1841. SGR_2423–2426 can explain the metabolism of Sop_n_s (Figure 6b). Although SGR_2427 is annotated as a hypothetical protein in the Kyoto Encyclopedia of Genes and Genomes (KEGG) database, a homologous protein from *Sanguibacter keddieii* has been found to be an SGL recently, and this phylogenetic group was classified into GH193, a newly established GH family (Nakajima et al. 2025). SGR_2422 is annotated as an acetyl‐esterase in the KEGG database, although this protein is not classified into any CE family (Lombard et al. 2010). SGR_2422 and SGR_2426 have no *N*‐terminal signal peptide. However, considering that SGR_2424–2425 (transmembrane subunits of ABC transporter) do not have an *N*‐terminal signal peptide either, SGR_2422 and SGR_2426 could be located extracellularly.

Genes encoding close homologs of SGR_2426 and SGR_2423 form gene clusters in several species, including some *Bifidobacterium* (Figure 6a). Among residues recognizing the Glc unit at subsite +1, Trp291 and Gln229 are conserved, and Met171 is also conserved as a hydrophobic residue (Figure 6c). Contrarily, Asn228 indirectly participating in recognition is not conserved. β‐1,2‐Glucans are produced in various microorganisms while the existence of Sop_n_s is reported mainly as sophorosides (Sop_2_‐linked compounds) (Kim et al. 2012). Although several gene clusters lack GH193 genes, five GH families of SGLs (GH144, GH162, GH192–194) were found, and their homologs are widely distributed among bacteria and fungi (Abe et al. 2017; Nakajima et al. 2025; Tanaka et al. 2019). Sop_n_s could be provided by these potential SGLs. Overall, SGR_2426 is a β‐glycosidase preferring β‐glucosides; more specifically, the enzyme is considered to be a β‐1,2‐glucosidase physiologically. Based on this, we speculate that *S. griseus* can metabolize (alkylated) Sop_n_s.

To understand structural determinants of the substrate specificity of the enzyme, subsite +1 in the SGR_2426r–Sop_2_ complex was compared with that in GH3 Lin1840 (Figure 5c). Lin1840 prefers Sop_2_ as a substrate but also shows comparable activity toward Lam_2_. The enzyme is considered to be involved in metabolism of β‐1,2‐glucans and/or Sop_n_s (Ishiguro et al. 2017; Nakajima et al. 2016). In the Lin1840–Sop_2_ complex, the Glc moiety at subsite +1 is sandwiched hydrophobically by aromatic rings of Trp271 and Tyr583, and forms multiple hydrogen bonds between the 3‐ and 4‐OH groups and Arg572 (Figure 5c, left). Meanwhile, in SGR_2426, Trp291 and Met181 are located perpendicularly to the six‐membered ring of the Glc moiety at the subsite +1, thereby forming a hydrophobic interaction (Figure 5c, right). Furthermore, only the 4‐hydroxy group forms a direct hydrogen bond with residue Gln229. These observations suggest that the mechanism of substrate preference is different between Lin1840 and SGR_2426.

**FIGURE 7:**
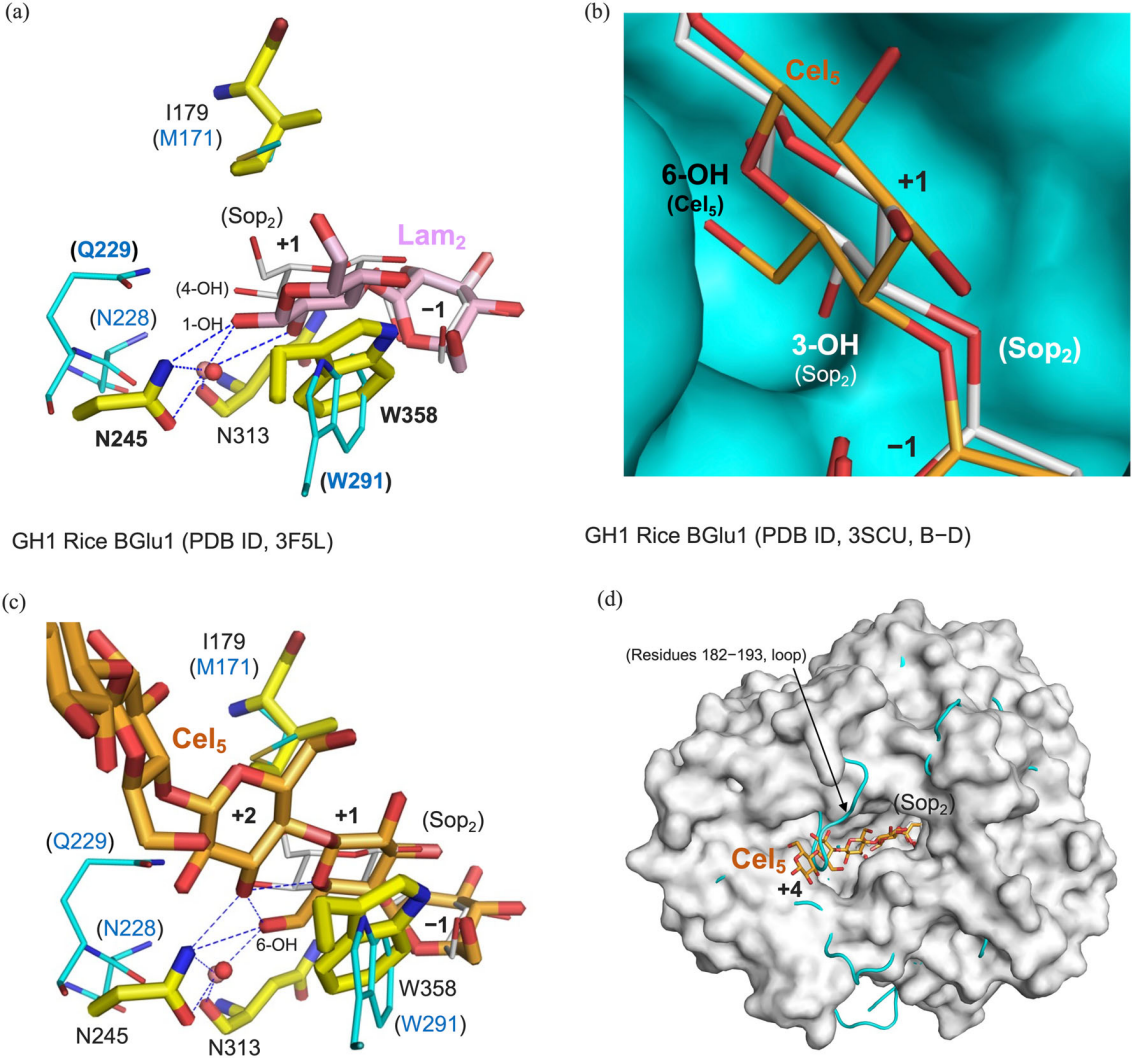
Comparison of binding modes of ligands with BGlu1 from *Oryza sativa*. (a–d) Superimposition of SGR_2426r–Sop_2_ complex with GH1 BGlu1–Lam_2_ (a) and BGlu1–Cel_5_ (b–d) complexes. Residues in SGR_2426r (PDB ID, 9U98) and BGlu1 are shown as cyan and yellow sticks, respectively. Lam_2_ and Cel_5_ are shown as pale pink and orange sticks, respectively. Sop_2_ in SGR_2426r is shown using thin white sticks. Parentheses used for residues and substrates represent labels for SGR_2426r. Hydrogen bonds are shown as blue dashed lines. Distances up to 3.5 Å are regarded as hydrogen bonds. (a) Residues indicated with bold letters are referred to in the main text. (b) SGR_2426r is shown as a cyan surface. Cel_5_ is shown as a thin stick. (d) Superimposition of SGR_2426r–Sop_2_ complex (cyan cartoon) with BGlu1–Cel_5_ complex (white surface).

Substrate recognition residues for subsite −1 are well conserved among GH1 enzymes (Figures 5 and S2). Thus, subsites +1 were compared using complexes with ligands binding both at subsites −1 and +1 among structurally characterized close homologs of SGR_2426 (Figures 5 and 7). Comparison with BGL167 and MeBglD2 brought up Trp291 and Gln229 as candidates for contributing substrate preference of SGR_2426. W291E did not obtain hydrolytic activity toward Gen_2_ (Table 1), although BGL167 shows hydrolytic activity toward Gen_2_ moiety in a substrate. This suggests a possibility that the aglycone in the substrate is important to act on Gen_2_ moiety for BGL167. It might also be possible that combination with structural changes at residues 228–229 in SGR_2426 is needed to obtain activity toward Gen_2_ because a single mutation at Gln229 did not add substrate preference for Gen_2_ (Table 1). Substitution of Gln229 to Asn decreased hydrolytic activity toward Sop_2_ while not so much toward Lam_2_ (Table 1). This suggests that Q229 highly contributes to substrate preference for Sop_2_.

Then, SGR_2426r was compared with another structurally available enzyme. BGlu1 from *O. sativa* is a GH1 enzyme homologous to SGR_2426 that shows wide substrate specificity, acting on Cel_n_s, Lam_n_s, and Sop_2_ (Opassiri et al. 2004). The Glc moieties at subsite +1 in SGR_2426r–Sop_2_ and BGlu1–Lam_2_ complexes superimpose well (Figure 7a). The 4‐OH group is recognized by Asn229, and the 3‐OH group faces the substrate pocket in the Sop_2_ molecule in SGR_2426r. The Lam_2_ molecule in BGlu1 also has hydroxy groups (1‐OH and 2‐OH) at similar positions to those of the Sop_2_ molecule (Figure 7a,b). Thus, it is unclear why SGR_2426 is highly specific for Sop_2_. Asn245 and Trp358, substrate recognition residues in BGlu1, are conserved in the primary structure of SGR_2426 (chemically for Asn245) (Figure S2). However, the position of Asn245 and the orientation of Trp358 in BGlu1 are apparently different from the corresponding residues in SGR_2426, which may result in their different affinity for Lam_2_ (Chuenchor et al. 2011).

The low activity of SGR_2426 toward Cel_2_ can be explained clearly, unlike in the case of Lam_2_. If the Glc moieties at subsite +1 in Cel_2_ and Sop_2_ are aligned in SGR_2426r, the 6‐hydroxy group in Cel_2_ potentially collides with the surface of SGR_2426r (Figure 7b) because there is insufficient space beyond 3‐OH of the Sop_2_ molecule at subsite +1 to accommodate the 6‐OH of Cel_2_. In the case of BGlu1, the position of the Glc moiety at subsite +1 in Cel_5_ deviates from the corresponding Glc moiety in SGR_2426r to form a hydrogen bond with Asn245, which enables the 6‐OH group in Cel_5_ to avoid such steric hindrance (Figure 7b–d). Residues 182–193 in SGR_2426r, narrowing the substrate pocket, are absent from BGlu1, which allows sufficient space for binding Cel_5_ (Figures 3, 7, and S2).

In the case of GH1 enzymes, when Sop_2_ is placed at subsites −1 and +1, the 1‐OH group at subsite +1 faces the solvent side. This implies GH1 originally has the potential to be a β‐1,2‐glucan‐associated enzyme. Given the broad diversity of the GH1 family, we actually found a GH1 enzyme for the metabolism of β‐1,2‐glucans and/or Sop_n_s. Our findings also reinforce that β‐1,2‐glucan‐associated enzymes are distributed widely in microorganisms, enhancing our understanding of the importance of β‐1,2‐glucans in nature.

## 4 MATERIALS AND METHODS

### 4.1 Materials

pNP‐β‐Glc was purchased from FUJIFILM Wako Chemical Corporation (Japan), and pNP‐β‐d‐Fuc and pNP‐β‐Gal from Nacalai Tesque, Inc. (Japan). Lam_n_s and Cel_2_ were purchased from Neogen (MI, USA), and Gen_2_ from Tokyo Chemical Industry (Japan). Sop_n_s were prepared according to the previous studies (Kobayashi et al. 2019; Nakajima et al. 2014; Nakajima et al. 2021).

### 4.2 Sequence analysis

Multiple sequence alignment was performed using MUSCLE (https://www.ebi.ac.uk/Tools/msa/muscle/) (Edgar 2004). A figure showing sequence alignment was prepared using ESPript 3.0 (https://espript.ibcp.fr/ESPript/cgi-bin/ESPript.cgi) (Robert and Gouet 2014).

### 4.3 Cloning, expression, and purification of recombinant SGR_2426 protein

The gene encoding SGR_2426 protein was amplified using *S. griseus* subsp. *griseus* (NBRC 13350) genomic DNA as the template. The genomic DNA was purchased from the National Institute of Technology and Evolution. Primer pair 5′–TTTAAGAAGGAGATATACATATGACACACACCCCTGCTTGGG–3′ and 5′–GTGGTGGTGGTGGTGGTGCTCGAGGGCTGCCGTGCGCGGGAGTTCG–3′ and PrimeSTAR Max DNA Polymerase (Takara, Japan) were used for PCR. The primer sequences were designed to fuse a His_6_‐tag at the *C*‐terminus of the protein. The PCR product was purified and then inserted into pET30a cut with restriction enzymes NdeI and XhoI (Takara) using the SLiCE method (Motohashi 2015). After the sequence of the inserted gene in the resulting plasmid was checked, the plasmid was transformed into *E. coli* BL21 (DE3). The transformant was seeded into Luria‐Bertani (LB) medium containing 30 μg/mL kanamycin and was cultured at 37°C overnight. The culture medium was seeded into LB medium containing the same antibiotic for large‐scale production of SGR_2426. After the culture medium was agitated vigorously until OD_600 nm_ reached 0.6–0.8, isopropyl β‐d‐1‐thiogalactopyranoside was added to 0.1 mM (final concentration) to induce production of SGR_2426r. The culture medium was kept agitated at the same velocity at 30°C overnight. The cells were collected by centrifugation at 5640*g* for approximately 5 min, and then suspended in 50 mM MOPS (pH 7.0) buffer. After the cells were disrupted by sonication, the suspension was centrifuged at 27,000*g* for 10 min. The supernatant was loaded onto a HisTrap™ FF crude column (5 mL; Cytiva, MA, USA) equilibrated with 50 mM Tris–HCl (pH 7.0) containing 500 mM NaCl (buffer A). The column was washed with buffer A containing 10 mM imidazole until most unbound proteins were removed. SGR_2426r was eluted with buffer A containing a 10–300 mM linear gradient of imidazole. The purity of the eluate was investigated by 10% SDS‐PAGE. The enzyme migrated at almost the same molecular weight as the theoretical molecular weight. The SGR_2426r solution was exchanged into 5 mM MOPS buffer (pH 7.0) by ultrafiltration (Vivaspin MWCO 10000, Sartorius, Germany). The concentration of SGR_2426r was calculated from absorbance at 280 nm. The theoretical molecular weight and extinction coefficient of SGR_2426r at 280 nm are 45691.255 Da and 103,735 mol^−1^ cm^−1^, respectively (Pace et al. 1995). No *N*‐terminal signal peptide was detected by SignalP5.0 analysis (Almagro Armenteros et al. 2019).

### 4.4 Temperature and pH profiles

To investigate the optimum pH for SGR_2426r activity, substrate solution (pNP‐β‐Glc, 95 μL) in various buffers was incubated at 30°C for 5 min, and then SGR_2426r solution (5 μL) at an appropriate concentration (1–3 μg) was added to start the reaction. The final concentrations of buffer and substrate were 50 mM and 5 mM, respectively. After the reaction mixtures were incubated at 30°C for 10 min, 20 μL sample was mixed with 180 μL of 0.2M Na_2_CO_3_ to stop the reaction. The mixture (175 μL) was put into a 96‐well microplate (EIA/RIA plate, 96‐well half area, Corning, NY, USA) and the sample absorbance was measured at 400 nm. pNP was used as a standard.

To investigate the optimum reaction temperature for the enzyme, the reaction procedure described above was applied at various temperatures with 50 mM MOPS (pH 7.0) as the buffer. For investigating pH and temperature stabilities, SGR_2426r solutions (0.011 mg/mL) were incubated for 1 h in 50 mM each buffer at 30°C, or 50 mM MOPS (pH 6.5) at each temperature. Enzyme activity assays were performed in 50 mM MOPS (pH 7.0) at 30°C for 10 min with 5 mM substrate. Experiments were performed in triplicate.

### 4.5 Kinetic analysis toward glucooligosaccharides

After substrate solutions (95 μL) in MOPS (pH 6.5, 50 mM) were incubated at 30°C for 5 min, SGR_2426r solution of an appropriate concentration (5 μL, 0.03–0.14 mg/mL) was added to start the reaction. When investigating substrate specificity for Lam_3–5_ as substrates, 45 μL of substrate solutions containing 5 mM substrate were used instead. Various substrate concentrations were adopted for the kinetic analysis. The reaction mixtures were incubated at 30°C for 10 min and then heated at 100°C for 5 min to terminate the reaction. The reaction mixtures (30 μL) were mixed with 170 μL of GOPOD solution (Neogen). After the samples were incubated at 45°C for 20 min, the absorbance of the samples was measured at 510 nm. d‐Glucose was used as a standard. Medians were plotted and the plots were regressed with the Michaelis–Menten equation by R. For substrates except for pNP‐β‐d‐Fuc and Sop_5_, the Michaelis–Menten equation *v* = *V*_max_ [S]/(*K*_m_ + [S]) was used. For pNP‐β‐d‐Fuc, the equation *v* = {(*V*_max_[S]/*K*_m_) + *V*_max2_[S]^2^/(*K*_m_ *K*_m2_)}/{1 + [S]/*K*_m_ + [S]^2^/(*K*_m_ *K*_m2_)} was used to adopt biphasic kinetics. For Sop_5_, the equation *v* = *V*_max_ [S]^n^/{*S*_0.5_^n^ + (1 + [S]/*K*_i_) [S]^n^} was used to take substrate inhibition into account. When calculating *V*_max_/*K*_m_, *V*_max2_/*K*_m2_ and *V*_max_/*S*_0.5_, *v* = (*V*_max_/*K*_m_) [S]/(1 + [S]/*K*_m_), *v* = {(*V*_max_/*K*_m_) [S] + (*V*_max2_/*K*_m2_) [S]^2^/*K*_m_}/{1 + [S]/*K*_m_ + [S]^2^/(*K*_m_ *K*_m2_)} and *v* = (*V*_max_/*S*_0.5_)[S]^n^/[{*S*_0.5_^n^ + (1 + [S]/*K*_i_) [S]^n^} *S*_0.5_], respectively, were used. The two values (*V*_max_/*K*_m_, *V*_max2_/*K*_m2_ or *V*_max_/*S*_0.5_, and *K*_m_, *K*_m2_ or *S*_0.5_) were set as variables. Linear regression through the origin was also used to confirm the plausibility of the values. Experiments were performed in triplicate.

### 4.6 Mutational analysis

E168Q, E316G, Q229N, and W291E mutants of SGR_2426r were constructed using PrimeSTAR Max DNA polymerase according to the manufacturer's instructions. The primer pairs used were 5′–ATCAACcaaCCGAACATGATCGCCGTG–3′ and 5′–GTTCGGttgGTTGATCGTGCAGACGTG–3′, 5′–GTCACGgggAACGGCATCGCCACCGCC–3′ and 5′–GCCGTTcccCGTGACGATCAGCGGGAC–3′, 5′–GCCAACaatGTCTACCAGGCGCTCCCC–3′ and 5′–GTAGACattGTTGGCGATGGTCCAGCC–3′, and 5′–ACGACGgaaGAGTACTACCCGACGGCG–3′ and 5′–GTACTCttcCGTCGTCAGGGTCCGCTC–3′, respectively. Replaced nucleotides are represented in small letters. Mutant enzymes were expressed and purified in the same way as wild‐type SGR_2426r.

### 4.7 Crystallography

Initial screening of crystallization conditions for wild‐type SGR_2426r was performed using PACT primer™ HT‐96 MD1‐36 and JCSG‐plus™ HT‐96 MD1‐40 (Molecular Dimensions, UK). Purified SGR_2426r (7.5 mg/mL, 0.5 or 1.0 μL) and reservoir solution (1.0 μL) were mixed in 96‐well CrystalQuick plates (Greiner Bio‐One, Germany) for sitting drop vapor diffusion with 70 μL reservoir solutions. After the plates were sealed, they were incubated at 20°C for several days until crystals were observed. Crystallization conditions were optimized using the wild‐type enzyme and the E318G mutant by hanging drop vapor diffusion using VDX plates (Hampton Research, Germany). The wild‐type enzyme (8.0 mg/mL, 1 μL) was mixed with 1 μL of reservoir solution containing 0.1M Bis‐Tris (pH 5.5), 0.2M ammonium acetate, and 15% (w/v) PEG 3350 (FUJIFILM Wako Chemical Corporation) for crystallization. In the case of the E318G mutant, 0.5 μL of 7.0 mg/mL enzyme solution was mixed with 0.5 μL of reservoir solution containing 0.1M Bis‐Tris propane (pH 6.5), 0.3M sodium formate, and 18% PEG 3350. Crystals of wild‐type SGR_2426r were soaked in reservoir solution containing 25% PEG 400 as a cryoprotectant. For the E318G mutant, the crystals were soaked in reservoir solution containing 25% PEG 200 as a cryoprotectant and 500 mM Sop_2_ for approximately 2 h. The crystal was cooled and then kept at 100 K in a nitrogen‐gas stream during data collection. X‐ray diffraction data were collected using a Pilatus detector at beamlines BL‐5A and NW‐12A at the Photon Factory, Tsukuba, Japan. The diffraction data set was processed by XDS (Kabsch 2010). The structure of a GH1 enzyme (PDB ID: 4R27) was used as a search model for molecular replacement by MOLREP (Vagin and Teplyakov 2010) to determine initial phases. Automated and manual model building were performed using ARP/wARP classic (Morris et al. 2002) and Coot, respectively (Emsley and Cowtan 2004). Refinement was performed using Refmac5 (Murshudov et al. 1997). The quality of the structures was checked using the wwPDB validation server (https://validate-rcsb-1.wwpdb.org/). Figures were prepared using PyMOL (DeLano Scientific, CA, USA).

### 4.8 MD simulation and RMSF analysis

The Chain A of 8KAP and Chain B of 9U98 were used for MD simulation as a ligand‐free and a complex structure of SGR_2426, respectively. The proteins and Sop_2_ were protonated and placed in a dodecahedral box. Box size was determined in order that all molecules were placed at least 1.5 nm from the box edges. The periodic boundary conditions were applied for all directions. The box was filled with water molecules. Sodium and chloride ions were added to each box to neutralize the total charge as well as to set the ion density to 10 mM. The AMBER ff14SB force field (Maier et al. 2015) and GLYCAM06 (Kirschner et al. 2008) were used to represent proteins and Sop_2_, respectively. The TIP3P model (Jorgensen et al. 1983) was used for water molecules. After energy minimization, the constant‐pressure and constant‐temperature (NPT) MD simulations for equilibration were performed at 1 bar and 300 K for 200 ps. Position restraints were applied to Cα atoms of the proteins and all heavy atoms of the Sop_2_ during equilibration. Three production runs for each system were performed for 500 ns at 300 K (total simulation time was 500 ns * 3 runs = 1.5 μs for the ligand‐free structure and the complex structure, respectively). The C‐rescale method and Parrinello‐Rahman method were used to maintain the pressure during the equilibrations and the production runs, respectively. The covalent bonds of hydrogen atoms were constrained using the LINCS algorithm (Hess et al. 1997), and the integration time step was 2.0 fs. The coordinates of atoms were output every 100 ps, and the total number of outputs was 15,000 frames (5000 frames * 3 runs). RMSF values of Cα atoms of proteins were calculated using the gmx rmsf command of GROMACS. The MD simulation and RMSF analysis were performed by using GROMACS 2022.4 (Abraham et al. 2015).

## AUTHOR CONTRIBUTIONS

**Haruto Kumakura:** Investigation; writing – review and editing; writing – original draft. **Sei Motouchi:** Investigation; writing – review and editing; funding acquisition. **Kaito Kobayashi:** Writing – review and editing; investigation. **Miyu Inoue:** Investigation; writing – review and editing. **Natsuki Kariuda:** Investigation; writing – review and editing. **Hiroyuki Nakai:** Conceptualization; resources; writing – review and editing. **Masahiro Nakajima:** Conceptualization; investigation; writing – original draft; writing – review and editing; funding acquisition; data curation.

## CONFLICT OF INTEREST STATEMENT

The authors declare no conflicts of interest.

## Supporting information


**Data S1.** Supporting Information.

**Data S2.** Supporting Information.

## Data Availability

The atomic coordinates and structure factors (codes 8KAP and 9U98) have been deposited in the PDB. The data that support the findings of this study are available from the corresponding author upon reasonable request.
