# Supplementary material for: A β‐1,2‐glucan‐associated glycoside hydrolase family 1 β‐glucosidase from Streptomyces griseus
Source: Protein Sci. 2025 Aug 21;34(9):e70255. doi: 10.1002/pro.70255 (PMC12369401; doi:10.1002/pro.70255)
Supplement: Supplementary file 1 — Data S1. Supporting Information. [file PRO-34-e70255-s001.docx]

**Table S1.** **Data collection and statistics**

| **Data set** | **Ligand-free (wild-type)** | **E318G-Sop_2_ complex** |
| --- | --- | --- |
| **Data collection** |  |  |
| Beamline | KEK NW-12A | KEK BL-5A |
| Space group | *P*2_1_2_1_2_1_ | *P*2_1_ |
| Unit cell parameters (Å) | *a* = 96.86  *b* = 97.76  *c* = 184.87 | *a* = 91.79  *b* = 100.18  *c* = 96.05  β = 102.87° |
| Resolution (Å)*^a^* | 48.43–2.20 (2.24–2.20) | 46.82–2.13 (2.17–2.13) |
| Total reflections*^a^* | 594223 (30764) | 318981 (15739) |
| Unique reflections*^a^* | 89749 (4548) | 93387 (4558) |
| Completeness (%)*^a^* | 100.0 (100.0) | 98.7 (98.2) |
| Multiplicity*^a^* | 6.6 (6.8) | 3.4 (3.5) |
| Mean *I*/σ(*I*)*^a^* | 8.8 (2.2) | 10.4 (2.1) |
| *R*_merge_ (%)*^a^* | 19.3 (90.2) | 8.8 (56.4) |
| *R*_pim_ (%)*^a^* | 12.2 (56.6) | 8.3 (51.5) |
| *CC*_1/2_*^a^* | (0. 685) | (0.637) |
| **Refinement** |  |  |
| Resolution (Å) | 46.261–2.20 | 46.82–2.13 |
| No. of reflections | 85285 | 88660 |
| No. of atoms | 12656 | 12837 |
| No. of water molecules | 302 | 530 |
| *R*_work_/*R*_free_ (%) | 23.2/25.7 | 18.1/22.8 |
| No. of asymmetric units | 4 | 4 |
| r.m.s.d. from ideal values |  |  |
| Bond lengths (Å) | 0.0022 | 0.0085 |
| Bond angles (°) | 0.8632 | 1.6657 |
| Average *B*-factors (Å^2^) |  |  |
| Protein (chain A/B/C/D) | 25.7/23.8/29.4/26.6 | 26.6/27.6/26.9/38.3 |
| Ligand |  |  |
| Sop_2_/Sop_2_/Sop_2_/Glc (chain A/B/C/D)  Sop_2_/Sop_2_ (chain A/C, subunit interface) |  | 44.5/39.8/45.3/47.5  36.9/38.0 |
| Solvent | 23.1 | 28.0 |
| Ramachandran plot (%) |  |  |
| Favored | 96.4 | 96.3 |
| Allowed | 3.4 | 3.6 |
| Outlier | 0.2 | 0.1 |
| **PDB entry** | 8KAP | 9U98 |

*^a^* Values in parentheses represent the highest resolution shell.


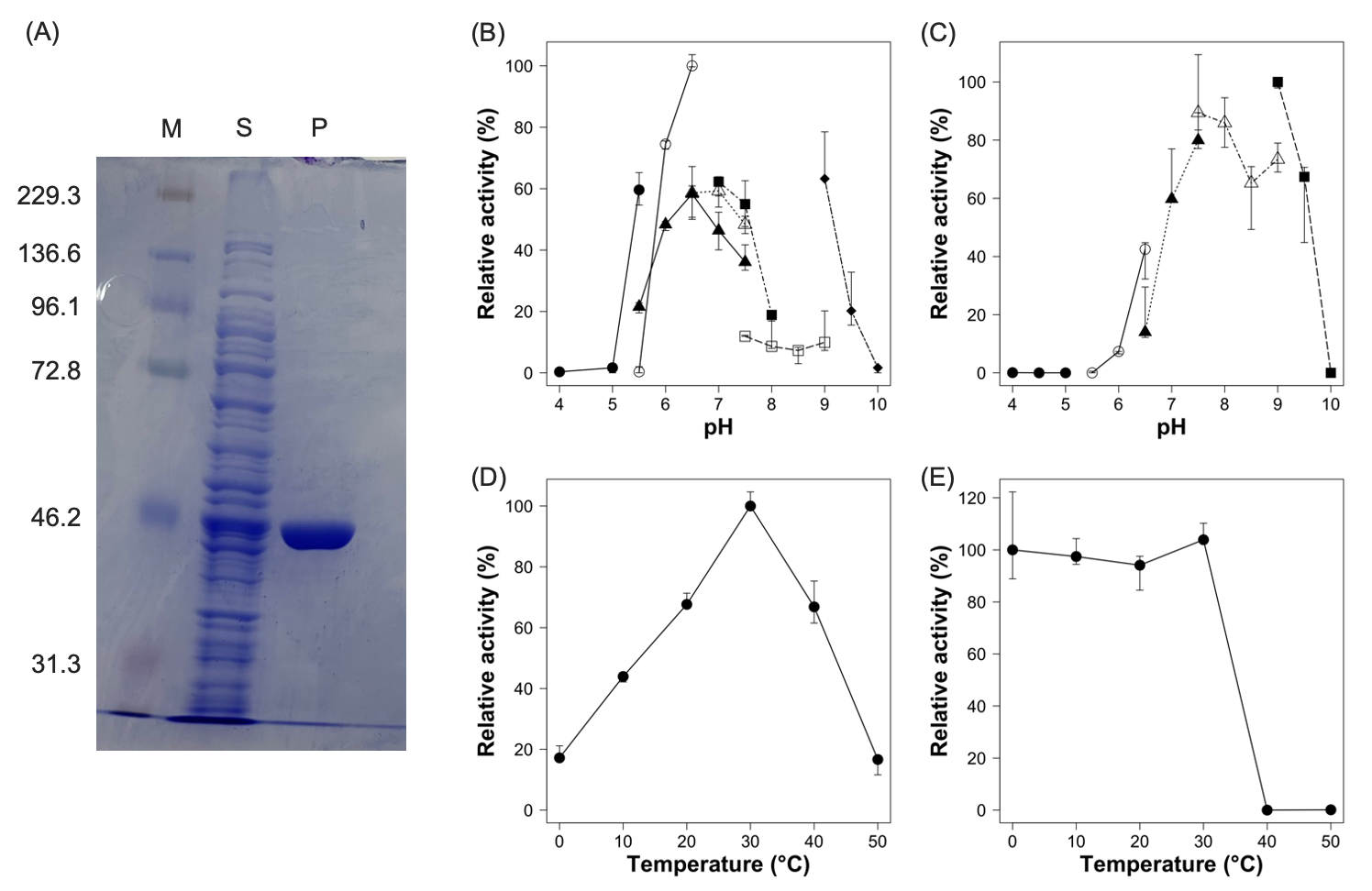


**Figure S1. General properties** **of SGR_2426r.**

(A) SDS-PAGE. M, protein marker (DynaMarker Protein MultiColor, BioDynamics Laboratory Inc., Japan); S, supernatant of cell extract; P, purified SGR_2426r. (B, C) Optimum reaction pH (B) and pH stability (C). Closed circles, sodium acetate; open circles, MES–NaOH; closed triangles, Bis-Tris–HCl; open triangles, MOPS–NaOH; closed squares, HEPES–NaOH; open squares, Tris–HCl; closed diamonds, glycine–NaOH. (D, E) Optimum reaction temperature (D) and thermal stability (E). Medians were plotted and the other data were used for error bars.


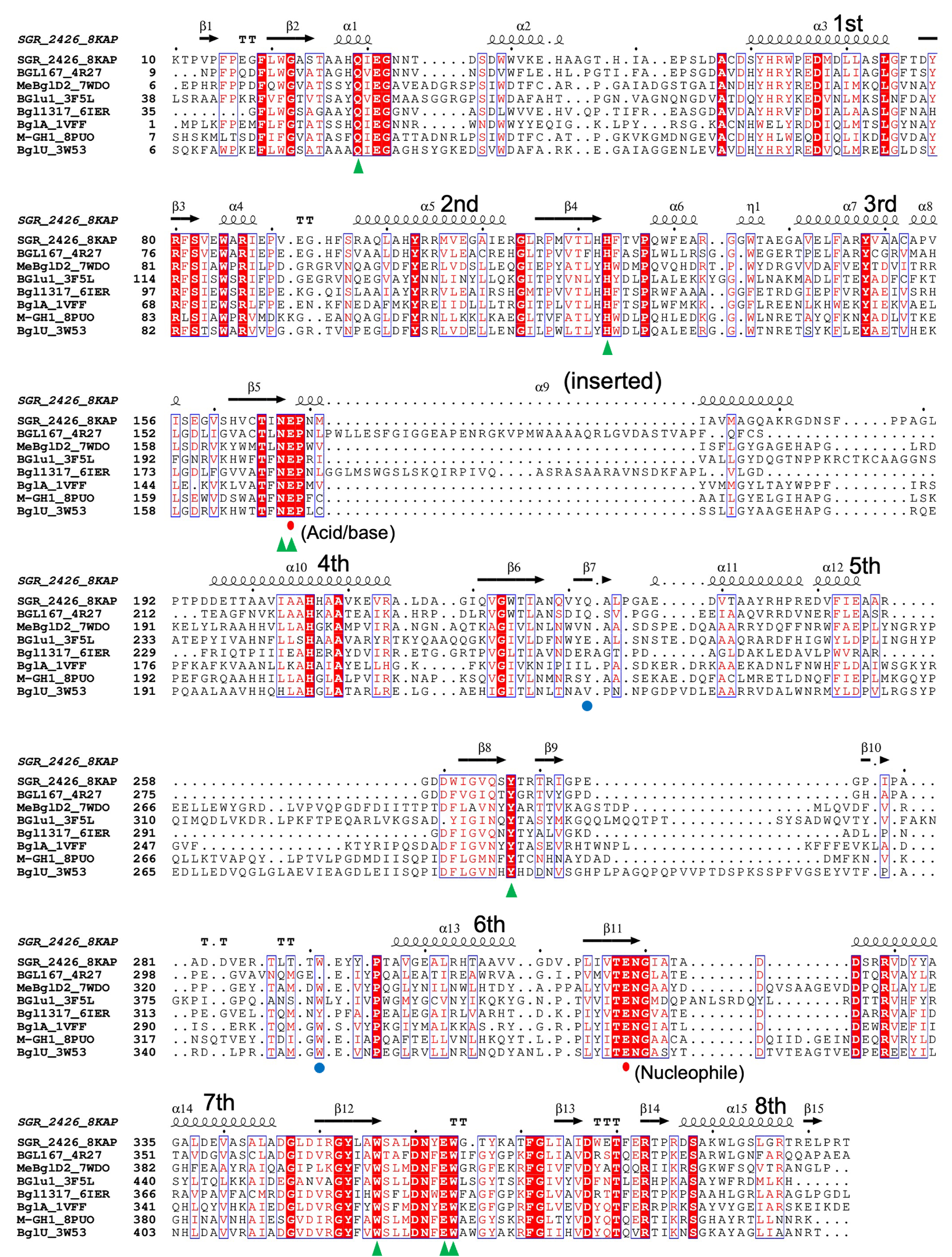


**Figure S2. Multiple sequence alignment of SGR_2426 and structurally** **available homologs.**

The enzymes BGL167 (PDB ID, 4R27), BGlu1 (3F5L), Bgl1317 (6IER), and BglA (1VFF), whose structures are used in the figures for discussion, rank among the top five hits in a KEGG database BLASTP search using SGR_2426 as the query. M-GH1 (8PUO) and BglU (3W53) are cold-adaptive GH1 BGLs. The bottom four enzymes have structures registered only in their ligand-free forms. The GenBank accession numbers are BGL167 (from *Microbacterium* sp. Gsoil167), AGA60135.1; MeBglD2 (from uncultured microorganism), BAV69317.1; BGlu1 (from *Oryza sativa* Japonica), CBD28469.1; Bgl1317 (from uncultured bacterium), QCG75868.1; BglA (from *Pyrococcus horikoshii*), BAA29440.1; M-GH1 (*Marinomonas* sp. BSi20584), PJE53865.1; BglU (*Micrococcus antarcticus*), ACM66669.1. Catalytic residues are labeled with red circles. Gln229 and Trp291 in SGR_2426 are labeled with blue circles. Recognition residues of a glucose unit at subsite −1 are labeled with green triangles. The ordinal numbers represent the order of α-helices in the (β/α)_8_-barrel from *N*-terminus of SGR_2426.


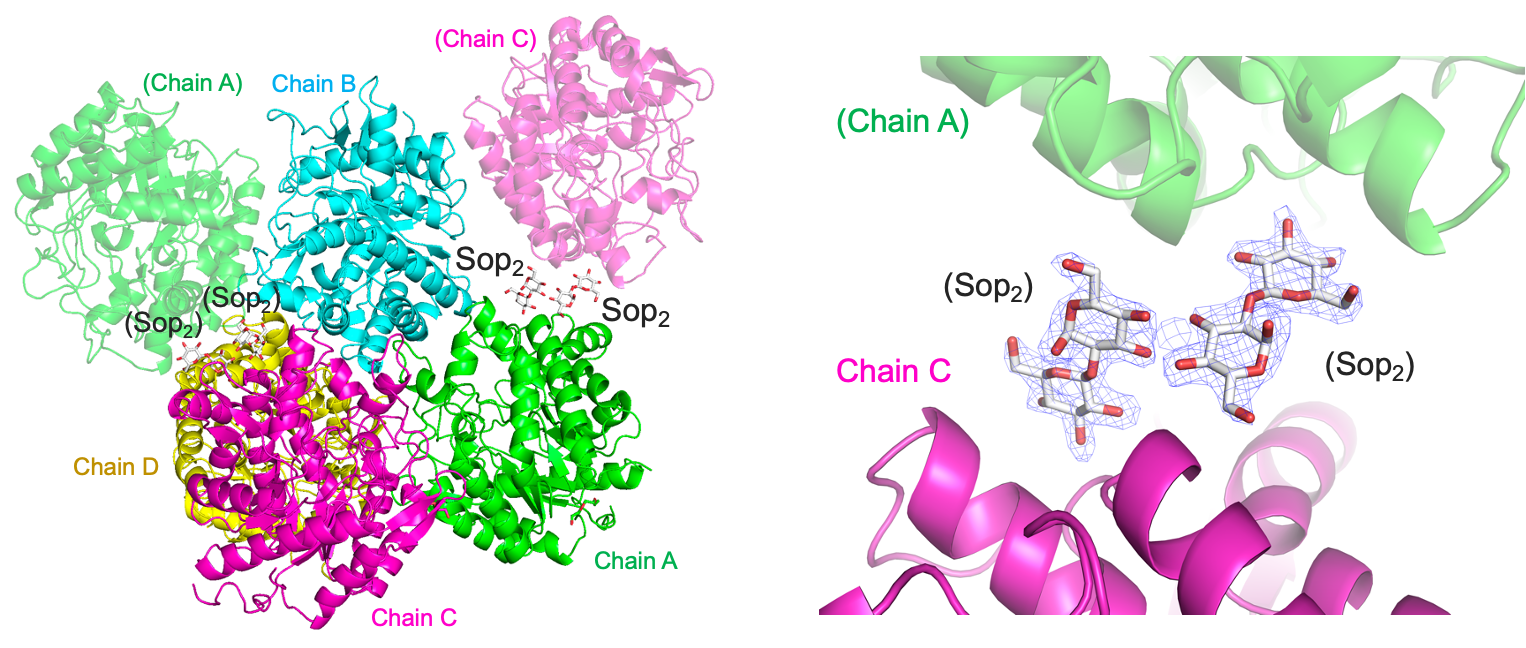


**Figure S3. Sop_2_** **molecules observed at the interface of chains.**

Chains A–D in the SGR_2426r–Sop_2_ complex are shown as green, cyan, magenta, and yellow cartoons, respectively. Sop_2_ molecules observed at the interface of chains are shown as white sticks. Chains of symmetry mates are shown semi-transparently. Parentheses represent symmetry mates. Parenthesis represents a symmetry mate and the labeled chain is shown semi-transparently. (left) Positions of Sop_2_ molecules at the interface in the overall structure. An arrow indicates the position of the Sop_2_ molecules. (right) Electron density of Sop_2_ molecules at the interface of the chains. This is an enlarged view of the Sop_2_ molecules in the left figure. A viewpoint is changed from the left figure.


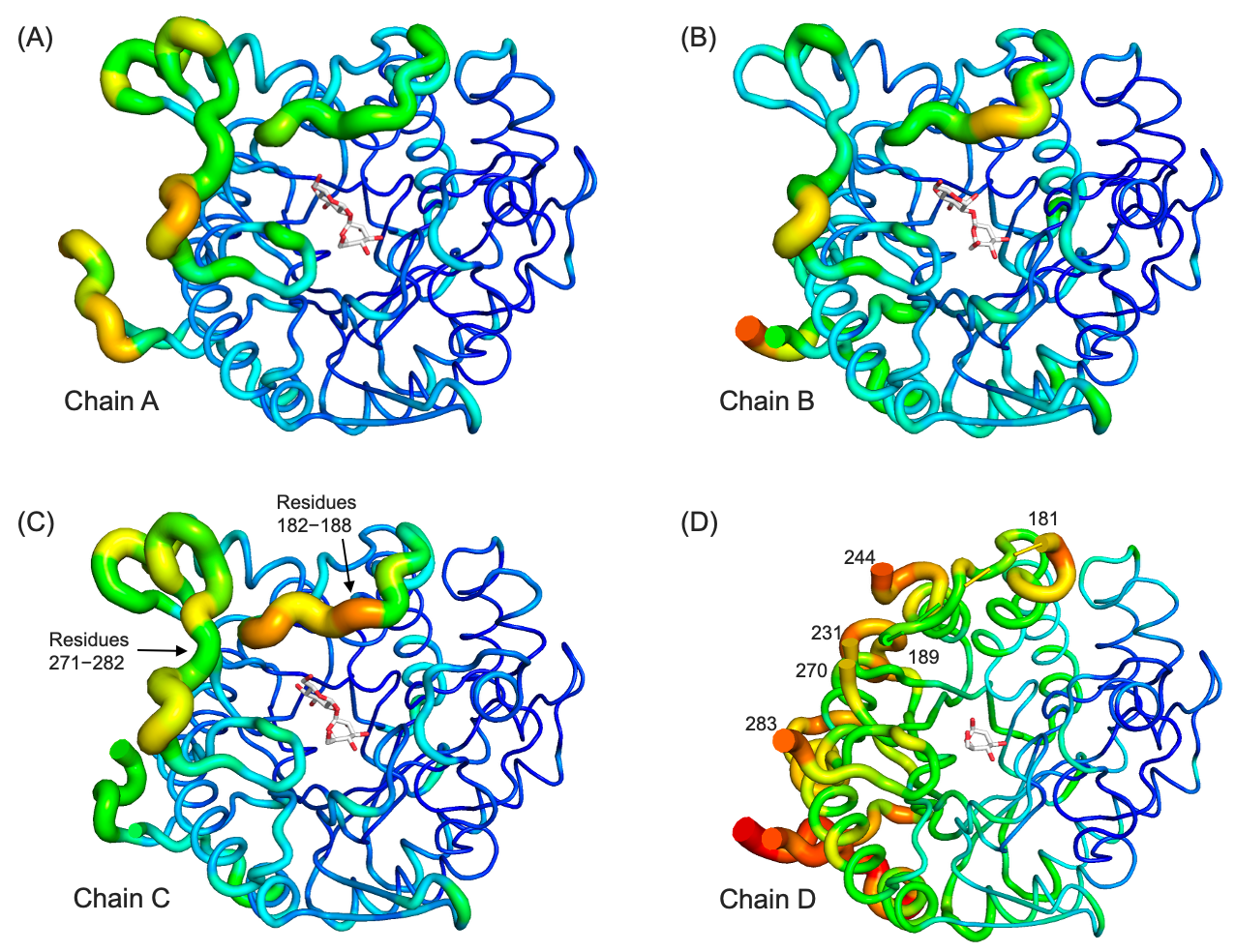


**Fig. S4. B-factors of SGR_2426r–Sop_2_ complex.**

B-factors are visualized by boldness and rainbow colors of main chains using PyMOL; a red, bold chain represents that the B-factor is high. Ligands are shown as white sticks. (C) Regions with large B-factors forming the substrate pocket are indicated with arrows and labels. Residue numbers correspond to disordered regions in (D). (D) Residue numbers at the edges of the disordered regions are shown.

**
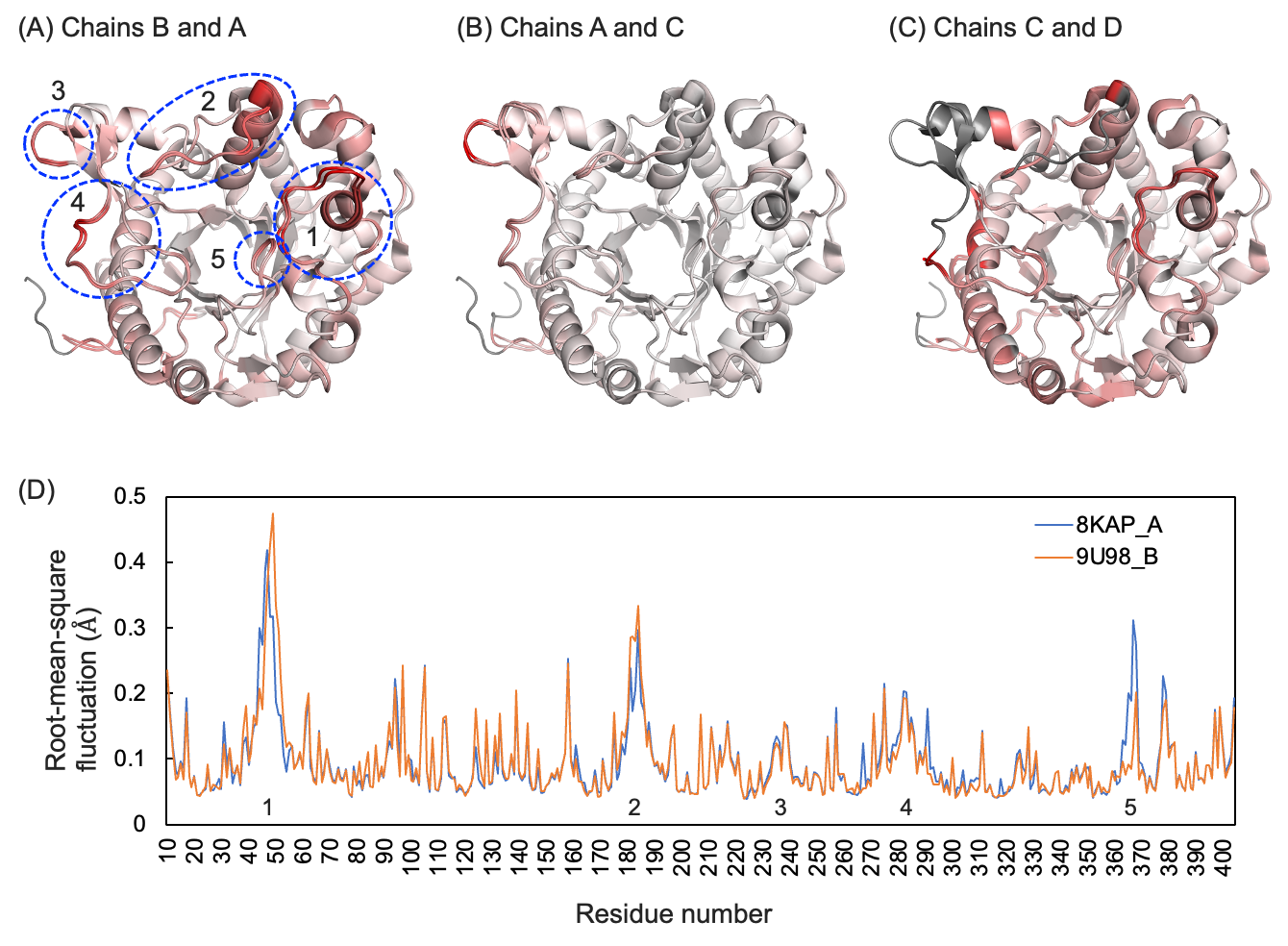
**

**Fig. S5. Deviation and fluctuation of SGR_2426r structures.**

(A–C) Superimposition of Chains B and A (A), A and C (B), and C and D (C). The extent of deviation is represented by a color gradient from white (small deviation) to red (large deviations). Regions that counterparts are disordered are colored in gray. Regions with large deviations are highlighted with blue dotted lines and are numbered from *N*-terminus. (D) MD simulation of SGR_2426r. The ligand-free structure (PDB ID, 8KAP, Chain A) and the complex with Sop_2_ (PDB ID, 9U98, Chain B) were used as the starting structures. The numbers above the x-axis represent the regions shown in (A).


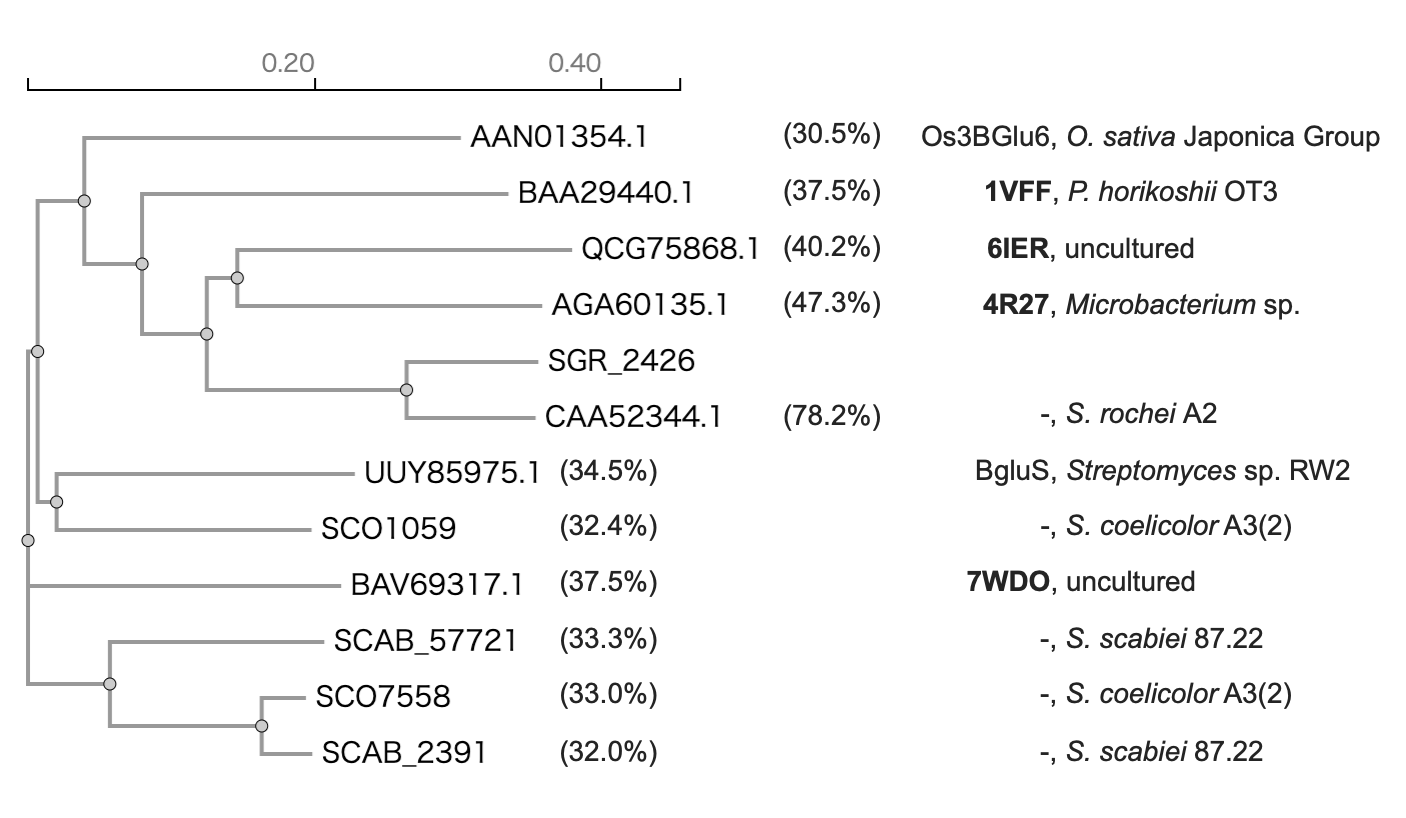


**Figure S6. Phylogenetic tree of GH1 enzymes.**

Multiple sequence alignment and visualization of a tree was performed using MUSCLE (68). Parentheses represent amino acid sequence identities with SGR_2426. Protein names or PDB IDs (bold letters), and organism names are shown on the right. The lengths of the branches are indicated by a scale above the tree.
